# Paclitaxel chemotherapy disrupts microbiota-enterohepatic bile acid metabolism in mice

**DOI:** 10.1080/19490976.2024.2410475

**Published:** 2024-10-01

**Authors:** Brett R. Loman, Zainab Alzoubi, Alexis J. Lynch, Robert M. Jaggers, Kelley Jordan, Corena V. Grant, Lynette K. Rogers, Leah M. Pyter, Michael T. Bailey

**Affiliations:** a Center for Microbial Pathogenesis, Abigail Wexner Research Institute at Nationwide Children’s Hospital, Columbus, OH, USA; b Division of Nutritional Sciences, University of Illinois at Urbana-Champaign, Urbana, IL, USA; c Department of Animal Sciences, University of Illinois at Urbana-Champaign, Urbana, IL, USA; d Institute for Behavioral Medicine Research, The Ohio State University, Columbus, OH, USA; e Center for Perinatal Research, Abigail Wexner Research Institute at Nationwide Children’s Hospital, Columbus, OH, USA; f Department of Pediatrics, The Ohio State University, Columbus, OH, USA; g Department of Psychiatry and Behavioral Health, The Ohio State University, Columbus, OH, USA; h Department of Neuroscience, The Ohio State University, Columbus, OH, USA

**Keywords:** Microbiome, gut-liver axis, cholestasis, endotoxin, malabsorption

## Abstract

Balanced interactions between the enteric microbiota and enterohepatic organs are essential to bile acid homeostasis, and thus normal gastrointestinal function. Disruption of these interactions by cancer treatment instigates bile acid malabsorption, leading to treatment delays, malnutrition, and decreased quality of life. However, the nature of chemotherapy-induced bile acid malabsorption remains poorly characterized with limited treatment options. Therefore, this study sought to characterize changes in hepatic, enteric, and microbial bile acid metabolism in a mouse model of chemotherapy-induced toxicity. Consistent with clinical bile acid malabsorption, chemotherapy increased fecal excretion of primary bile acids and water, while diminishing microbiome diversity, secondary bile acid formation, and small intestinal bile acid signaling. We identified new contributors to pathology of bile acid malabsorption in the forms of lipopolysaccharide-induced cholestasis and colonic crypt hyperplasia from reduced secondary bile acid signaling. Chemotherapy reduced markers of hepatic bile flow and bile acid synthesis, elevated markers of fibrosis and endotoxemia, and altered transcription of genes at all stages of bile acid metabolism. Primary hepatocytes exposed to lipopolysaccharide (but not chemotherapy) replicated chemotherapy-induced transcriptional differences, while gut microbial transplant into germ-free mice replicated very few differences. In the colon, chemotherapy-altered bile acid profiles (particularly higher tauromuricholic acid and lower hyodeoxycholic acid) coincided with crypt hyperplasia. Exposing primary colonoids to hyodeoxycholic acid reduced proliferation, while gut microbiota transplant enhanced proliferation. Together, these investigations reveal complex involvement of the entire microbiota-enterohepatic axis in chemotherapy-induced bile acid malabsorption. Interventions to reduce hepatic lipopolysaccharide exposure and enhance microbial bile acid metabolism represent promising co-therapies to cancer treatment.

## Introduction

Cancer and chemotherapy are associated with numerous gastrointestinal side effects, including diarrhea, nausea, and gut barrier disruption.^1,2^ These symptoms often lead to delays or cessation of treatment, may persist for decades, and significantly compromise quality of life.^3,4^ Diarrhea is particularly common during chemotherapy, where it is reported in up to 80% of patients.^5^ Bile acid malabsorption (BAM) is a major cause of diarrhea following chemotherapy, as up to half of patients receiving chemotherapy are diagnosed with BAM.^6^ BAM is clinically characterized by greater fecal excretion of primary BA, water, and energy, and diminished gut-liver signaling.^7^ Patients diagnosed with BAM traditionally have limited treatment options: either low-fat, low-fiber diets or prescription bile acid sequestrants. These therapies provide limited efficacy and have low patient adherence due to low palatability, abdominal discomfort, and constipation. A recent attempt to establish clinical practice guidelines for gastrointestinal mucositis (an underlying contributor to BAM) resulted in the creation of no new guidelines due to inadequate or conflicting evidence.^8^ Furthermore, BAM is likely underdiagnosed due to the preferential attention given to efficacy of cancer therapy (tumor elimination) and restricted availability of standardized diagnostic tests. Together, this highlights the need for alternative diagnostic and therapeutic options that target the underlying causes of BAM, not just its symptoms.

Effective treatment of BAM is hindered by its poorly characterized pathophysiology. Broadly, BAM can occur as a consequence of elevated BA synthesis and excretion by the liver, or reduced capacity to reabsorb BA in the terminal small intestine. Chemotherapy-induced BAM is presumed to occur following chemotherapy-induced enterotoxicity of the rapidly-dividing ileal epithelium. This is reasonable, given that the ileum is the primary site of BA absorption, chemotherapy induces apoptosis of rapidly proliferating cells like those of the small intestine, and surgical resection of the ileum induces BAM.^6,9^ This usually coincides with inflammation and ulceration across the gastrointestinal mucosa, known as mucositis. Loss of tissue integrity, underlying immune responses, and disruption to the enteric microbiota likely contribute to diarrheal symptoms.^10^ However, contributions beyond the small intestine are rarely considered. The liver has great influence over BA homeostasis as the primary site of BA synthesis and excretion. The colon is highly responsive to BA escaping the ileum, influencing electrolyte and fluid absorption, motility, barrier function, and cellular proliferation.^11^ Finally, gut microbes perform extensive modifications to bile acids throughout the gastrointestinal tract, changing their physical and biological signaling properties.^12^ Thus, the liver, colon, and enteric microbiota likely influence chemotherapy-induced BAM, and require investigation.

The purpose of this study was to characterize the effects of chemotherapy on the microbiota-enterohepatic axis with particular respect to BA metabolism and malabsorption. Herein, we demonstrate in a clinically relevant mouse model that the entire microbiota-enterohepatic axis is disrupted by chemotherapy. BAM is not influenced solely by reduced BA absorption in the ileum, but also hepatic lipopolysaccharide exposure and reduced bacterial BA transformation by colonic bacteria. Finally, we demonstrate that these effects can be partially recapitulated by transfer of gut microbes from chemotherapy-treated to germ-free mice, establishing gut barrier function and metabolism of the enteric microbiota as important targets in the development and treatment of BAM.

## Methods

### Animals, chemotherapy, and sample collection

All experimental protocols were approved by Institutional Animal Care and Use Committee at The Ohio State University. Singly housed, female, 7–8 week old BALB/c mice (Charles River, Wilmington, MA, USA) were acclimated to a 14:10 light:dark cycle in a temperature-controlled vivarium (22°C) 1 week prior to treatment.

Mice received 100 μL intraperitoneal injections of either 30 mg/kg paclitaxel chemotherapy (Chemo) (Sigma-Aldrich, St. Louis, MO, USA), which is primarily implemented in the treatment of female reproductive cancers^13^ or vehicle control (Vehicle) as previously described.^14^ This represents approximately 80% of an i.v. dose as administered to patients (FDA 2005) and based on a prior experiment.^14^ Six total doses were administered every other day during the light phase. Across the 15 days of the study period, mice received injections on days 2, 4, 6, 8, 10, and 12, followed by sacrifice on day 15. Paclitaxel is a taxane chemotherapeutic that stabilizes microtubule formation in rapidly dividing cells (preventing cell division and inducing apoptosis), which preferentially accumulates in the intestine even over target tumor tissue.^15^ Additionally, taxane treatment is associated with a high incidence of gastrointestinal toxicity including colitis, intestinal venous thromboses, and altered intestinal microbial populations,^3,16^ making it a suitable model for studying chemotherapy-microbiota-host interactions.

**Figure 1.**
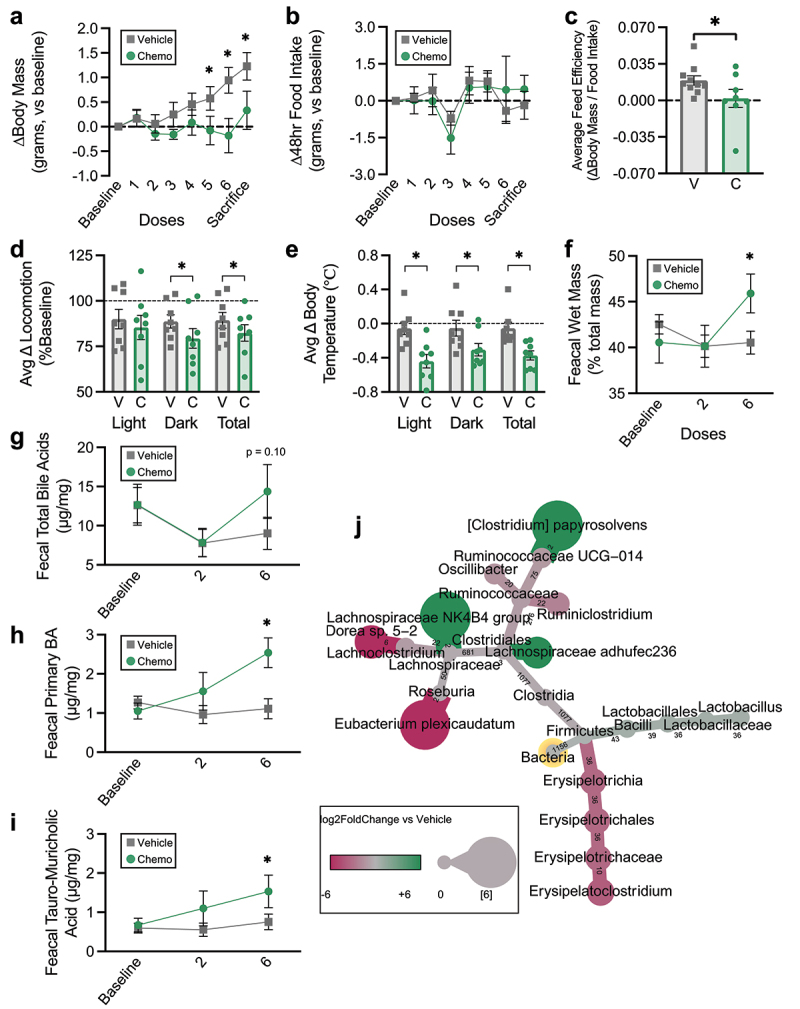
Chemotherapy induces weight stagnation, bile acid malabsorption, and altered fecal bacteriome composition. (a) Body mass changes from baseline. (b) Changes in 48 hour food intake from baseline. (c) Average feed efficiency during the study period. (dD) average light cycle, dark cycle, and total locomotion changes from baseline. (e) Average light cycle, dark cycle, and total body temperature changes from baseline. (f) Fecal wet mass changes from baseline. (g) Fecal total BA excretion throughout the study period. (h) Fecal primary BA excretion throughout the study period. (i) Fecal TMCA excretion throughout the study period. (j) Heat tree displaying differences in fecal 16S rRNA gene amplicon profiles in Chemo vs vehicle the day following the final dose. Nodes display log2FoldChange and edges display number of amplicon sequence variants composing each node. Baseline measurements are the average of two days prior to treatment. (a-c) *N* = 18 (vehicle *n* = 10, chemo *n* = 8), (d-e) *N* = 16 (vehicle *n* = 8, chemo *n* = 8), (f-j) *N* ≤ 18 (vehicle *n* = 8-10, chemo *n* = 5-8), * *p* < 0.05.

All data were collected in a single cohort (Vehicle *n* = 10, Chemo *n* = 8), except that collected via electronic-transmitters (Figure 1b-c; Vehicle *n* = 8, Chemo *n* = 8). In the primary cohort, body mass and 48-hour food intake was recorded for two days before treatment (baseline) and upon dosing until sacrifice. Fecal samples were collected at baseline (day 1) and the days following the second and final doses (days 5 and 13). Euthanasia occurred three days following the final dose of chemotherapy, during the dark cycle via CO_2_ asphyxiation. Samples were collected using autoclaved instruments and tubes. Samples were immediately frozen on dry ice then stored at −80°C until analyzed. Other data for these animals, including microbial beta diversity and crypt morphology of the colon have been reported elsewhere.^14^

In the electronic-transmitters cohort, electronic-transmitters (G2 E-mitters, Starr Life Sciences, Oakmont, PA) were implanted into the peritoneal cavity as previously described to continuously assess core body temperature and home-cage locomotion.^17^ Starting with 48 hours of baseline, body temperature and number of locomotor counts were averaged for both the light and dark phases of each day.

### Bile acids

BAs were quantified using a modified method described by Si, et al., designed to extract and separate a wide range of BA (i.e. conjugated, unconjugated, primary, and secondary) from complex biological samples.^18^ BA were extracted from fecal samples, intestinal contents, and liver tissue for quantification via HPLC. Samples were homogenized in 10 mm potassium phosphate pH 6.0, centrifuged at 12,000 RPM for 20 min at 4°C, and the supernatant was removed for subsequent extractions. The supernatant was extracted twice with acetonitrile, dried under nitrogen gas at 60°C, then reconstituted in methanol for HPLC analysis.

Prepared samples were analyzed on a Shimadzu Nexera XR HPLC equipped with UV detection. A Zorbax-SB C18 column (150 × 4.6 mm id., 5 mm) was used with mobile phase A 0.01 M potassium phosphate, pH 2.6 and mobile phase B 100% acetonitrile A linear gradient was used for elution (0–2 min 25% B, 2–17 min 25–33% B, 17–37 min 85% B, and 37–40 min 85% B). The flow rate was 1.0 mL/min and the detection wavelength was set at 200 nm. The column temperature was maintained at 40°C and the injection volumes were 25 mL.

BA standards came from the following sources: tauro-cholic acid (TCA), cholic acid (CA), tauro-chenodeoxycholic acid (TCDCA), chenodeoxycholic acid (CDCA), tauro-deoxycholic acid (TDCA), deoxycholic acid (DCA), tauro-α-muricholic acid (α-TMCA), tauro-β-muricholic acid (β-TMCA), α-muricholic acid (αMCA), β-muricholic acid (βMCA), tauro-ursodeoxycholic acid (TUDCA), lithocholic acid (LCA), hyocholic acid (HCA), and hyodexoycholic acid (HDCA) from Cayman Chemical Company, Ann Arbor, MI; tauro-lithocholic acid (TLCA), ursodeoxycholic acid (UDCA), and tauro-hyodeoxycholic acid (THDCA) from MilliporeSigma, Burlington, MA. Standard curves were constructed with concentrations ranging from 0.01 to 1 mm. Peaks for α-TMCA and β-TMCA could not be separated, so they are reported collectively as TMCA, a problem that is not unique to our study.^19^ Sample concentrations were derived from the experimentally derived standard curve.

### Microbiome

Feces and intestinal contents were sent to The Environmental Sample Preparation and Sequencing Facility at Argonne National Laboratory for DNA extraction, library preparation, and high-throughput sequencing. Paired-end (250 nt forward and reverse) sequences of the V4 hypervariable region of the 16S rRNA gene (515F-806 R) were generated on Illumina MiSeq. Quantitative Insights into Microbial Ecology 2 (QIIME2) was utilized for amplicon processing, quality control with DADA2, downstream taxonomic assignment using the SILVAv132 database, and diversity analyses.^20,21^ Fecal samples were rarefied to 8,800 reads/sample (2 Vehicle and 1 Chemo samples excluded) and intestinal content samples were rarefied to 12,800 reads/sample (0 duodenum, 1 jejunum [1 Vehicle], 0 ileum, 1 cecum [1 Vehicle], 0 proximal colon, and 3 distal colon [3 Vehicle] samples excluded due to low read counts). Sequencing data are available at https://www.ncbi.nlm.nih.gov/bioproject/PRJNA750499.

Differential abundance of bacterial taxa was determined utilizing DESeq2, and heat trees were plotted via Metacoder in R.^22–24^ False discovery rate correction was not applied for this exploratory analysis. Log2FoldChanges of statistically significant differentially abundant taxa at all taxonomic levels were plotted as on heat trees, utilizing node size to denote magnitude of differential abundance, node color to signify directionality of differential abundance (i.e. positive or negative), and numeric labeling of edges to denote number of amplicon sequence variants (ASVs) contributing to each node.

To elucidate bacterial taxa that were associated with differences in colonic BA profiles, bacterial differentials were built in Songbird with treatment group, intestinal segment, and the interaction effect in the model.^25^ This model produced lower error and loss compared to the null model, and was utilized in subsequent analyses. Visualization of differentials and construction of logRatios were conducted in Qurro.^26^ The initial logRatio was chosen to include the top and bottom 1% of features (by model feature rankings constructed in Songbird), and then refined to include all ASVs from genera identified in the initial logRatio that have been associated with BA metabolism. The final logRatio was correlated to the ratio of tauro-muricholic acid to hyodeoxycholic acid to determine relationship strength.

### Histological staining

Small sections of tissue (approximately half of the hepatic medial lobe, half of the spleen, and 1 cm of each intestinal segment) were fixed in methacarn overnight, paraffin-embedded, and sectioned at 4 μm for histological analyses. Slides were imaged with the Aperio AT Turbo system, and visualized and measured in Aperio ImageScope software v12.3.2.8013 (Leica Biosystems, Wetzlar, Germany).

Hal’s bile stain was performed on liver tissue using Hall’s Bile Stain Kit according to manufacturer instructions (StatLab Medical Products, McKinney, TX). Positively stained area and intensity were quantified using the Positive Pixel Count v9 Analysis in Aperio ImageScope, with the following modified settings: Compression Quality = 95, Hue Value = 0.15, Hue Width = 0.15, Color Saturation Threshold = 0.1, Iwp(Low) = Ip (High) = 140, Ip(Low) = Isp(High) = 100.

Trichrome stain was performed on liver tissue using a commercially available kit according to the manufacturer instructions (Abcam, Cambridge, UK). Positively stained collagen was measured and expressed as percent of total tissue area utilizing imageJ.

Tissue from all intestinal segments was stained hematoxylin and eosin for morphological analysis of the crypt-villus axis. Crypt depth, crypt width, villus height, and villus width were measured for 8–10 well-oriented structures per sample (all within-sample standard error <10% of the mean) across ≥3 sections. Small intestinal absorptive surface area was calculated as in Kisielinski et al. 2002, utilizing a formula with crypt width, villus height, villus width, which was multiplied by segment length.^27^

KI-67 immunohistochemistry was initiated by deparaffinization and rehydration in xylene, 50% xylene/50% ethanol, and a series of graded ethanol/PBS baths. Antigen retrieval was performed in a water bath at 99°C in citrate buffer, pH 6.0 (Vector Laboratories, Burlingame, CA) for 30 minutes, followed by 10 minutes cool-down under running deionized water. Tissue was encircled with a hydrophobic pen, then covered with blocking buffer containing: 0.3% Triton X-100, 1% bovine serum albumin, and 10% normal goat serum (MilliporeSigma, Burlington, MA). The mouse-anti-KI-67 antibody (ThermoFisher Scientific, #14-5698-82) was diluted 1:150 in blocking buffer, and tissue was incubated overnight at 4°C on a shaker. The goat-anti-mouse antibody (ThermoFisher Scientific, # A-11077) was diluted 1:200 in blocking buffer, and tissue was incubated for one hour at room temperature on a shaker in the dark. Nuclei were stained with DAPI diluted 1:1000 in PBS for 15 minutes on a shaker in the dark. Between each antibody/DAPI exposure, slides were washed three times with PBS for 5 minutes each. Slides were mounted with Prolong Gold (ThermoFisher Scientific, Waltham, MA) and allowed to cure in the dark overnight prior to imaging. Given the fluorescence of goblet cells (excluding measurement of total fluorescence intensity) and the susceptibility of highly proliferative cells (i.e. KI-67 positive cells) to chemotherapy, distance of the highest KI-67^+^ nucleus from the crypt base was utilized as in Berger et al. 2018.^28^

### ELISAs

Total protein was extracted from liver and colon tissues via homogenization in RIPA Lysis and Extraction Buffer with Halt™ Protease Inhibitor Cocktail and quantified via Pierce™ BCA Protein Assay Kit according to manufacturer instructions (ThermoFisher Scientific, Waltham, MA). Lipopolysaccharide (LPS) binding protein was quantified from liver (Hycult Biotech, Uden, the Netherlands), and TNF and IL-6 were quantified from liver, colon, and primary CD11b^+^ leukocyte supernatant (V-PLEX proinflammatory panel 1 mouse kit, Meso Scale Diagnostics, Rockville, Maryland).

### Gene expression

Total RNA was extracted from liver, spleen, intestine, primary hepatocytes, and primary colonoids using Trizol™ reagent (Life Technologies Corporation, Carlsbad, CA, USA) per manufacturer instructions. cDNA was synthesized from RNA utilizing the High-Capacity cDNA Reverse Transcription Kit (Applied Biosystems, Foster City, CA, USA) per manufacturer instructions. Expression of specific genes was conducted by qPCR using Power SYBR Green PCR Master Mix (Applied Biosystems, Foster City, CA, USA) and normalized with the relative standard curve method with eukaryotic translation elongation factor 2 (*Eef2*) as the reference gene.^29^ Primers used in this study are listed in supplementary table S1.

### Primary hepatocyte culture

Primary hepatocytes were isolated from female BALB/c mice as modified from Seglen 1976.^30^ Briefly, mice were CO_2_ asphyxiated, and immediately perfused with pre-warmed (37°C) Kreb’s buffer with 1 mm EDTA and the portal vein clamped. Once the entire liver lightened in color, mice were perfused with pre-warmed Kreb’s buffer with 150 μM CaCl_2_. The excised liver was thoroughly minced with surgical scissors, triturated with a pipette, poured through a 70 μM nylon strainer, and washed three times with Kreb’s buffer. Hepatocytes (~3.2 × 10^5^ cells/well) were cultured on Biocoat Collagen I-coated Microplates (Corning, Corning, NY) in Dulbecco’s Modified Eagle Medium (DMEM; ATCC, Manassas, VA) supplemented with 10% fetal bovine serum (FBS), ITS liquid media supplement, 10 μg/mL Epidermal Growth Factor, 10 μg/mL dexamethasone, 0.5 mg/mL nicotinamide, and antibiotic/antimycotic.

Hepatocytes were cultured for two days before exposure to LPS and/or paclitaxel chemotherapy for two hours. Paclitaxel was delivered at a concentration of 80 μg/mL to mimic hepatic concentrations *in-vivo* after a single intraperitoneal injection of 36 mg/kg body mass.^31^ LPS was delivered at 1 μg/mL (L4524, MilliporeSigma, Burlington, MA). Hepatocytes were assayed via the CellTiter 96® Aqueous One Solution Cell Proliferation Assay (MTS Assay; Promega, Madison, WI) at the end of their respective incubations per manufacturer instructions. Hepatocytes were isolated three days after chemotherapy/LPS exposure to mimic *in-vivo* outcomes captured in this study.

### Primary colonoid culture

Primary colonoids were isolated from BALB/c mice utilizing an in-house protocol. Female BALB/c mice were CO_2_ asphyxiated, and the colon was immediately excised and placed into ice cold PBS. Contents were flushed using a gavage needle and tissue was transferred into DMEM with 10% FBS and antibiotic/antimycotic on ice and cut into ≤2 mm pieces. Tissue pieces were transferred into digestion solution (Hank’s Balanced Salt Solution, 5 mm EDTA, 5% FBS, 1 mm DTT, and antibiotic/antimycotic) and gently rotated for 20 minutes at 37°C. After vortexing for 10 seconds, the solution was passed through a 100 μm cell strainer. Tissue caught in cell strainer was digested, vortexed, and strained again. The combined flow-through was centrifuged at 400 g for 10 minutes, the cell pellet was resuspended in DMEM (without FBS), centrifuged again, and resuspended in DMEM prior to crypt counting. After resuspending crypts to an appropriate volume in complete IntestiCult™ Organoid Growth Medium (Human; StemCell Technologies, Cambridge, MA), 4 units of Matrigel (Corning, Corning, NY) per 1 unit of media, and plated 200–300 crypts per well in 50 μL media-Matrigel suspension with an additional 500 μL of complete organoid growth medium once Matrigel was set. Colonoids were passaged twice, and underwent colonoid differentiation according to StemCell protocol prior to BA and immunogen exposure.

Colonoids were exposed 0.1 mg/mL TMCA based on colonic concentrations in the Chemo group, 0.1 mg/mL HDCA based on colonic concentrations in the Vehicle group, 1 μg/mL LPS, and/or 1 μg/mL zymosan (Z4250, MilliporeSigma, Burlington, MA) for 48 hours in a 2 × 2 × 2 × 2 factorial design prior to collection for gene expression.

### Gut microbiota transplant

Procedures and other data collected for gut microbiota transplant mice have been reported elsewhere.^32^ Briefly, contents of the cecum and proximal colon of chemotherapy or vehicle-treated mice (treated with the same chemotherapy protocol as conventional animals in the current study) were diluted in anaerobic Schaedler broth (30 mg/mL), held briefly on ice, and gavaged intragastrically to germ-free mice a single time (100 uL). Gnotobiotic mice were maintained on an Allentown Sentry SPP IVC rack system, which utilizes positive pressure cages with HEPA filtered incoming air, and extract filtered outgoing air. All food, water, and absorptive paper bedding were autoclaved prior to utilization. Mice were left undisturbed under sterile conditions for one week prior to sacrifice and tissue collection.

## Results

### Paclitaxel chemotherapy induces weight stagnation, bile acid malabsorption, and altered fecal bacteriome composition

To explore effects of chemotherapy on microbiota-enterohepatic BA metabolism, we employed an established model of paclitaxel chemotherapy in mice. Chemo mice experienced stagnated body mass growth that was lower than Vehicle control mice by the fifth dose (Figure 1a). Notably, this difference in body mass was not related to differences in food consumption, implying that Chemo mice may suffer nutrient malabsorption (Figure 1b). This was corroborated by lower average feed efficiency (body mass change to food mass consumption ratio) in Chemo mice across the study period (Figure 1c). To further characterize factors potentially contributing to body mass differences, we investigated other potential sources of energy loss. Chemo mice exhibited decreased total locomotion (Figure 1d), particularly in the dark cycle, and lower body temperatures during the study period (Figure 1e).

Nutrient, water, and BA malabsorption are linked in patients with BAM. Chemo mice produced noticeably softer, wetter stools following repeated doses, indicative of malabsorption. Indeed, fecal wet mass was increased in Chemo mice by the final dose (Figure 1f). Total fecal BA displayed some inter-dose and intra-subject variability but tended to increase in Chemo mice by the end of treatment (Figure 1g). However, fecal BA was marked by an elevation in primary BA in Chemo mice (Figure 1h), particularly the main primary BA in rodents, tauro-muricholic acid (TMCA) (Figure 1i). Since primary BA are typically metabolized by the colonic microbiota, we hypothesized that this increase in conjugated BA would be accompanied by altered fecal microbiomes. The microbiomes of the two treatment groups were deemed equivalent at baseline, given that the only difference in composition was a mildly higher relative abundance of *Clostridiales Family XIII* in Chemo mice (0.1%±0.02 vs 0.02%±0.02%, nine sample counts = 0). Despite this, there were apparent differences in the fecal microbiomes of Chemo vs Vehicle mice by the end of treatment in the phylum Firmicutes, including a higher relative abundance of *Lactobacillus*, and variable abundances within the family *Clostridiales* (not *Clostridiales Family XIII*) (Figure 1j).^33^

### Paclitaxel chemotherapy disrupts hepatic bile acid homeostasis

**Figure 2.**
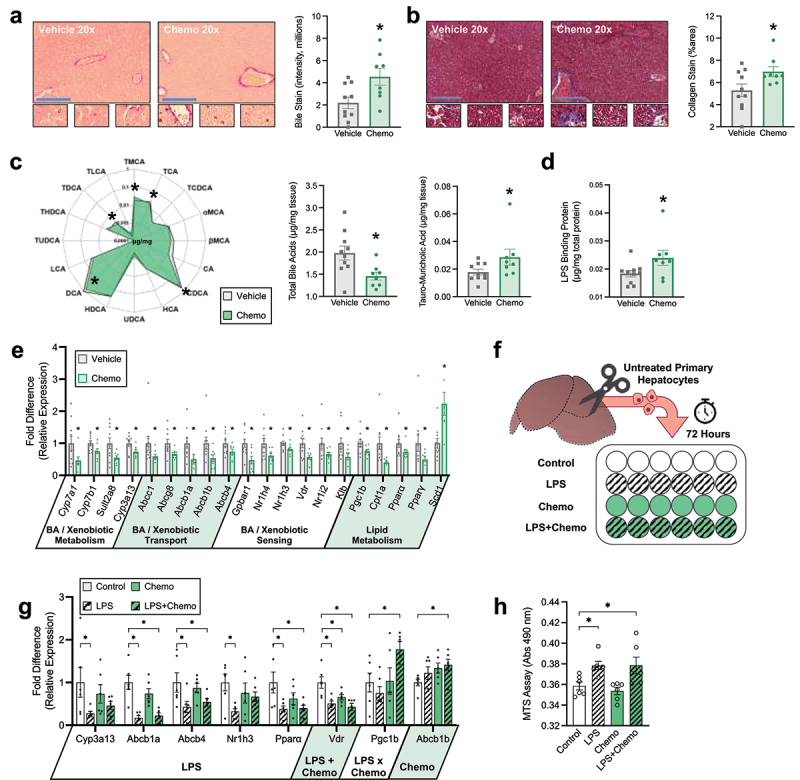
Chemotherapy disrupts hepatic bile acid homeostasis. (a) Hepatic Hal’s bile stain for bilirubin (marker of bile accumulation). Positive staining is green to brown in color. (b) Hepatic trichrome stain for collagen (marker of fibrosis). Positive staining is blue. (c) Radar plot displays hepatic concentration of all BA species measured, bar plots display hepatic total BA, and TMCA. (d) Hepatic LPS binding protein concentration. (e) Hepatic relative expression of genes involved in BA and xenobiotic metabolism, BA transport, BA sensing, and lipid metabolism; functional roles of genes are displayed under the x axis. (f) Graphic representation of primary hepatocyte culture and exposure study design. (g) MTS assay for hepatocyte viability. (h) Gene expression from *in vitro* exposure of primary hepatocytes to LPS and Chemo; main effects of exposures are displayed under the x axis. Blue scale bars = 100 μm, small zoomed sections = 60× magnification. (a-e) *N* = 18 (vehicle *n* = 10, chemo *n* = 8), (g-h) *N* = 24 (*n* = 6/group), *p < 0.05.

Given the stool consistency and higher BA concentrations consistent with BAM in this model, and scarcity of information regarding hepatic involvement in BAM, livers were analyzed for histological, metabolic, and transcriptional alterations contributing to BA homeostasis. Hall’s Bile Stain (an assay that stains bilirubin) was used to visualize hepatic bile accumulation, and trichrome stain was used to evaluate hepatic fibrosis. Chemo livers exhibited higher bile accumulation and fibrosis compared to Vehicle (Figure 2a-b). Additionally, hepatic BA profiles were altered by Chemo, where total BA were diminished, but TMCA was elevated (Figure 2c, supplementary table S2). Elevated bilirubin is an indicator of cholestasis (impaired bile flow), which can occur from bacteria-driven endotoxemia.^34,35^ Chemo livers contained higher lipopolysaccharide binding protein (LBP), an indicator of lipopolysaccharide (endotoxin) (LPS) exposure (Figure 2d).

Chemo altered hepatic transcription of genes related to the metabolism, transport, and sensing of BA, xenobiotics, and lipids (Figure 2e). Expression of rate limiting enzymes from both classical and alternative BA synthesis pathways, *Cyp7a1* and *Cyp7b1*, were reduced in Chemo livers, corroborating lower hepatic BA concentration. There were no differences in the expression of bile acid-CoA:amino acid N-acyltransferase (*Baat*), the enzyme that conjugates BA (Vehicle = 1.29 ± 0.09 vs Chemo = 1.19 ± 0.09 [mean ± SE] *p* = 0.42). Additionally, expression of primary BA-sulfating enzyme, *Sult2a8*, and primary paclitaxel chemotherapy-hydroxylating enzyme, *Cyp3a13*, were lower with Chemo. Expression of several transporters that regulate the bile flow to biliary excretion, *Abcg8*, *Abcb1a, Abcb1b*, and *Abcb4*, and BA into circulation, *Abcc1*, were diminished in Chemo livers, corroborating higher bile staining in these animals. Additionally, Chemo livers exhibited dampened expression of BA receptors that regulate expression of BA homeostatic genes and link BA signaling to lipid metabolism. As anticipated, this was associated with lower transcripts related to lipid metabolism, with the exception of *Scd1*, which was elevated.

Next, we determined if either LPS or chemotherapy exposure directly affects BA synthesis and transport by exposing primary hepatocytes to physiologically relevant concentrations of both *in vitro* (Figure 2f). LPS, but not chemotherapy, diminished expression of similar genes in primary hepatocytes as observed in Chemo livers with a few caveats (Figure 2g). Specifically, *Abcb1b* expression was not altered by LPS, but increased as a main effect of chemotherapy, *Vdr* was decreased by both LPS and chemotherapy, and *Pgc1b* was induced by the combination of LPS and chemotherapy. Expression of other genes altered in vivo were not altered by Chemo or LPS in vitro (data not shown). Notably, changes in hepatocyte transcription were not due to the loss of hepatocyte viability (Figure 2h).

### Paclitaxel chemotherapy reduces small intestinal bile acid absorptive capacity

**Figure 3.**
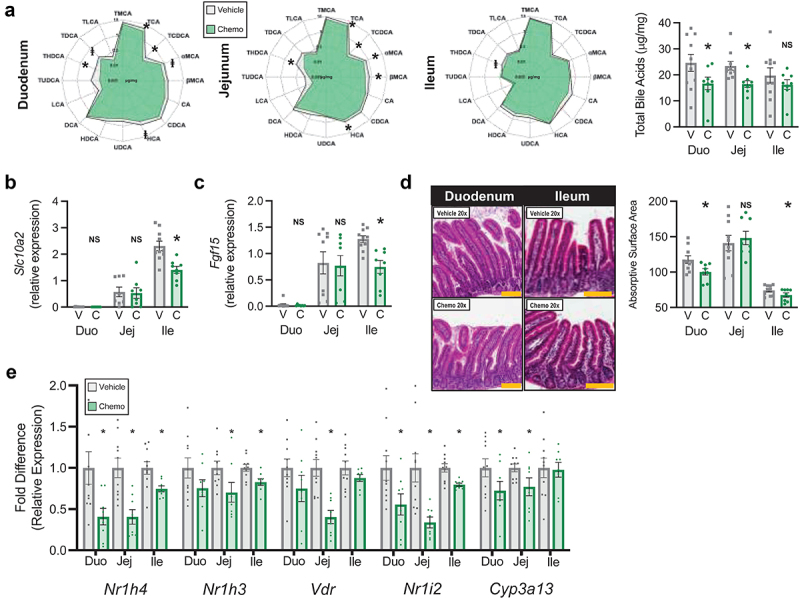
Chemotherapy reduces small intestinal bile acid absorptive capacity. (a) Radar plots display concentration of all luminal BA species measured, bar plot displays total luminal bile acids. (b) Relative expression of small intestinal *Slc10a2* (primary bile acid transporter). (c) Relative expression of small intestinal *Fgf15* (responsive to absorbed BA). (d) Small intestinal representative images and absorptive surface area. (e) Small intestinal expression of BA receptors and chemo-metabolizing gene *Cyp3a13*. *N* = 18 (vehicle *n* = 10, chemo *n* = 8), *p < 0.05, NS=not significant. V=vehicle, C=chemotherapy, Duo=duodenum, Jej=jejunum, Ile=ileum. Gold scale bar = 100 μm.

To determine if our model mimics the clinical paradigm of chemotherapy-induced enterotoxicity as a contributor to BAM, we turned our attention to metabolomics, histology, and transcription of the small intestine. As expected as a consequence of cholestasis, total BA were reduced in duodenal and jejunal contents of Chemo mice, but were similar in the ileum (Figure 3a, supplementary table S2). Intestinal absorption is governed by both anatomical structure and functional transporters. Expression of the chief BA transporter *Slc10a2*, BA absorption-responsive *Fgf15*, and absorptive surface area were reduced by Chemo in the ileum, the primary site of BA absorption (Figure 3b-d, **supplementary table S3**). Notably, expression of basolateral BA transporter heterodimers *Slc51*α and *Slc51*β were not affected by Chemo (**supplementary table S3**). Additionally, Chemo dampened expression of BA receptors *Nr1h4, Nr1h3, Nr1i2*, and *Vdr* as well as Chemo-detoxifying enzyme *Cyp3a13* throughout the small intestine (Figure 3e).

### Paclitaxel chemotherapy-altered bile acid profiles are related to colonic crypt hyperplasia

**Figure 4.**
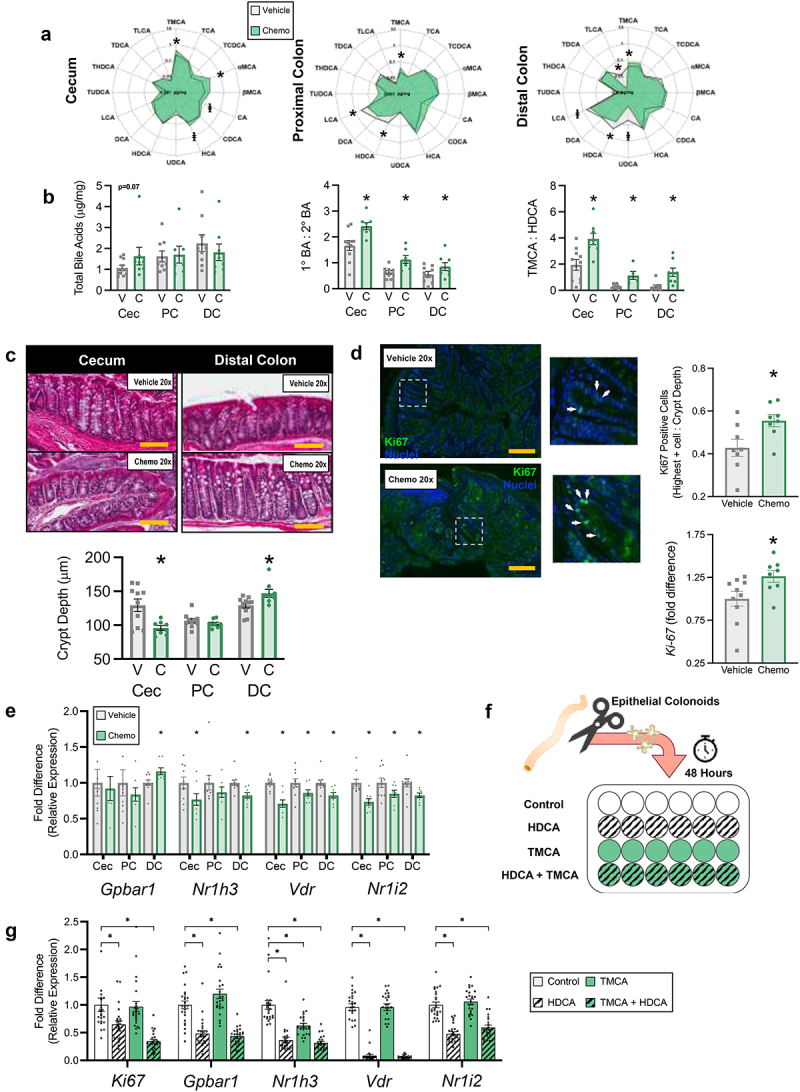
Chemotherapy-altered bile acid profiles are related to colonic crypt hyperplasia. (a) Radar plots display concentration of all luminal BA species measured. (b) Total luminal BA, ratios of luminal primary BA to secondary BA, and primary BA TMCA to secondary BA HDCA. (c) Representative images and distal intestinal crypt depths. (d) Representative images and immunofluorescent staining and gene expression of the proliferation marker ki-67. Golden scale bar = 100 μm and white arrows indicate ki-67 positive nuclei. (e) Distal intestinal relative expression of BA receptors. (f) Graphic representation of colonoid culture and exposure study design. (g) Gene expression from *in vitro* exposure of primary colonoids to physiological concentrations of HDCA and TMCA. (a-e) *N* = 18 (vehicle *n* = 10, chemo *n* = 8), (g) *N* = 96 (*n* = 24/group), *p < 0.05. V=vehicle, C=chemotherapy, Cec=cecum, PC=proximal colon, DC=distal colon, BA=BA, TMCA=tauro-muricholic acid, HDCA=hyodeoxycholic acid.

With BAM likely occurring in the Chemo small intestine, we next investigated metabolomics, histology, and transcription of the distal intestine. BA profiles were altered across the cecal, proximal colonic, and distal colonic contents of Chemo mice (Figure 4a-b, supplementary table S2), with an increased ratio of primary BA to secondary BA. This ratio was exaggerated for the primary BA TMCA vs its microbial metabolite and secondary BA HDCA.

Similar to the small intestine, the distal intestine of Chemo mice exhibited segment-dependent morphological changes. While crypt depth was diminished in the cecum and unaltered in the proximal colon, crypt depth of the distal colon was enhanced in Chemo mice (Figure 4c). To explain this phenomenon we performed fluorescent immunohistochemistry and qPCR of proliferation marker Ki-67. Indeed, Ki-67 fluorescence and expression were enhanced in Chemo mice (Figure 4d). Additionally, BA receptor expression was suppressed by Chemo in the distal intestine, except *Gpbar1*, which was enhanced in the distal colon (Figure 4e).

Next we determined if altered BA profiles, particularly TMCA:HDCA, could explain altered morphology and transcription in the distal colon by exposing primary colonoids to BA concentrations similar to *in vivo* observations (specifically, the concentration of TMCA in Chemo mice, and the concentration of HDCA in Vehicle mice). Mimicking these observations, colonoids exposed to HDCA (but not TMCA) exhibited diminished expression of *Ki-67* and *Gpbar1*, while TMCA reduced expression of *Nr1h3* (Figure 4o-q). However, HDCA also diminished expression of *Vdr* and *Nr1i2* (Figure 4r-s).

### Paclitaxel chemotherapy-induced alterations of the intestinal microbiome are related to altered bile acid profiles

**Figure 5.**
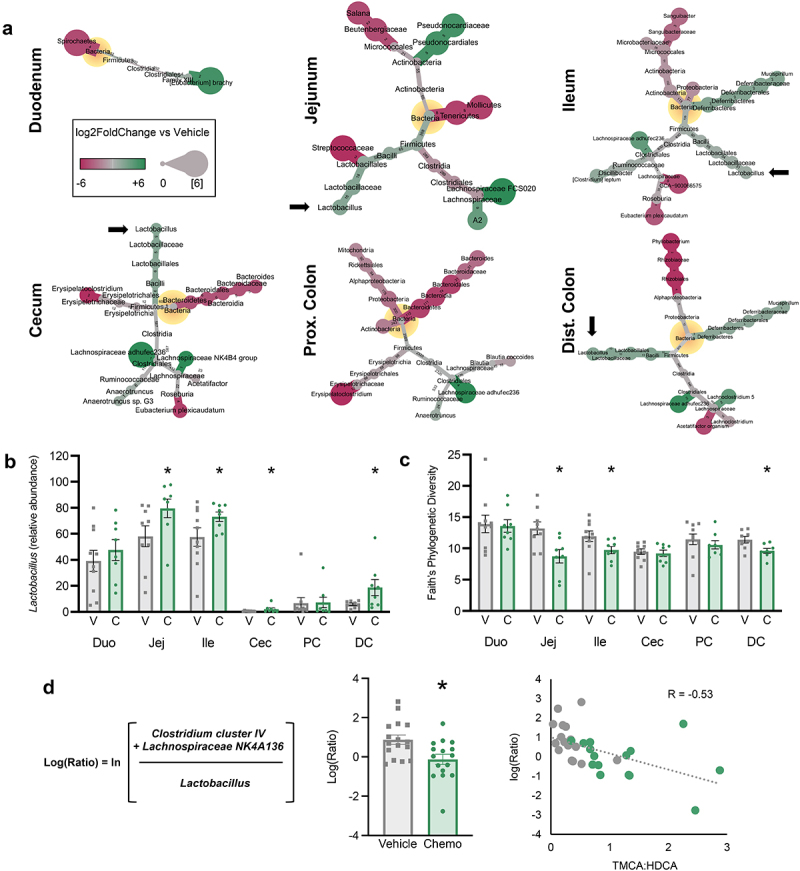
Chemotherapy-induced alterations of the intestinal microbiome are related to altered BA profiles. (a) Heat trees display significant differences in 16S rRNA gene amplicon profiles in Chemo vs vehicle. Nodes displaying log2FoldChange and edges displaying number of amplicon sequence variants composing each node. Black arrows emphasize *Lactobacillus*. (b) Relative abundance of *lactobacillus* in intestinal contents. (c) Alpha diversity (Faith’s phylogenetic diversity). (d) A bacterial differential [i.e. log(ratio) of the relative abundances of specific taxa] generated via Songbird and correlation to ratio of TMCA to HDCA. *N* ≤ 18 (vehicle *n* = 7-10, chemo *n* = 8), *p < 0.05, V=vehicle, C=chemotherapy, PC=proximal colon, DC=distal colon, TMCA=tauro-muricholic acid, HDCA=hyodeoxycholic acid.

Elevated ratio of primary BA to secondary BA led us to hypothesize that Chemo altered the intestinal microbiome, since secondary BA are strictly bacterial metabolites. Based upon 16S rRNA gene amplicon sequencing, the Chemo bacteriome was altered in every intestinal segment (Figure 5a). Higher relative abundance of *Lactobacillus* was a characteristic change, occurring in 4 out of 6 segments (Figure 5b), which coincided with lower alpha diversity (Faith’s Phylogenetic Diversity) (Figure 5c). While other compositional alterations were not as consistent across segments, other notable shifts occuring in more than one segment as a result of Chemo include enhanced *Lachnospiraceae adhufec236* (ileum, proximal colon, and distal colon), *Anaerotruncus* (cecum and proximal colon), and *Mucispirillum* (ileum and distal colon); but diminished *Micrococcales* (jejunum and ileum), *Eubacterium plexicaudatum* (ileum and cecum), *Erysipelotrichaceae* (cecum and proximal colon), *Bacteroides* (cecum and proximal colon), and *Acetatifactor* (cecum and distal colon).

To ascertain which bacteria could be responsible for the conversion of TMCA to HDCA, we identified relevant bacterial differentials via Songbird. The logRatio of *Clostridium cluster IV* and *Lachnospiraceae NK4A136* vs *Lactobacillus* was lower across Chemo colons, and inversely related to TMCA:HDCA (Figure 5l-n). This implied that enhanced relative abundance of *Lactobacillus* was associated with higher TMCA, and that diminished relative abundance of *Clostridium cluster IV* and *Lachnospiraceae NK4A136* was associated with lower HDCA.

### Gut microbiota transplant from paclitaxel chemotherapy-treated to germ-free mice recapitulates many phenotypic markers of bile acid dysregulation

To further elucidate the role of the enteric microbiota in chemotherapy-induced BA dysregulation, we employed gut microbiota transplant (GMT) from either chemotherapy-treated (Chemo-GMT) or vehicle control (Vehicle-GMT) donors into germ-free mice. The liver, ileum, and colon of GMT mice were examined for the same primary endpoints demonstrated in conventional animals.

**Figure 6.**
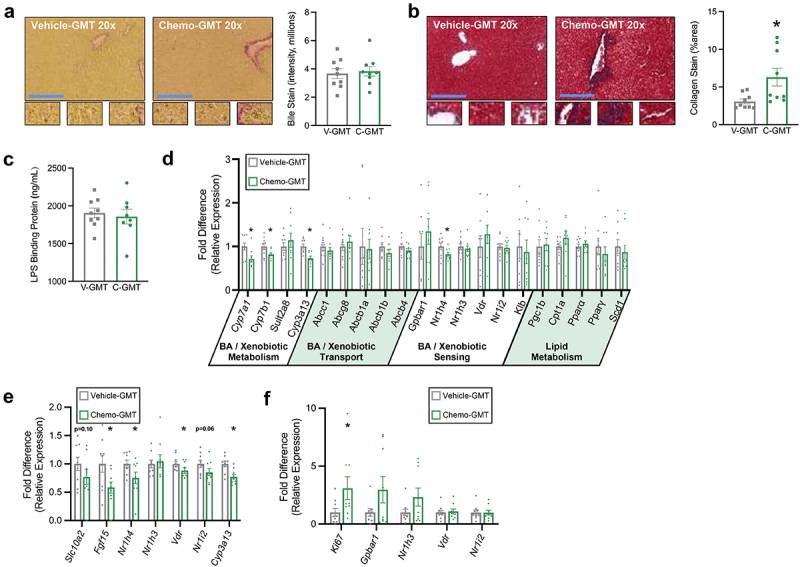
Gut microbiota transplant from chemotherapy-treated to germ-free mice recapitulates many phenotypic markers of bile acid dysregulation. (a) Hepatic Hal’s bile stain for bilirubin (marker of bile accumulation). Positive staining is green to brown in color. (b) Hepatic trichrome stain for collagen (marker of fibrosis). Positive staining is blue. (c) Serum LPS binding protein concentration. (d) Hepatic relative expression of genes involved in BA and xenobiotic metabolism, BA transport, BA sensing, and lipid metabolism. (e) Ileal expression of BA receptors and chemo-metabolizing gene *Cyp3a13*. (f) Distal colonic expression of BA receptors and proliferation marker *Ki67*. (a-f) *N* = 18 (vehicle-gmt *n* = 9, chemo-gmt *n* = 9), *p < 0.05. GMT = gut microbiota transplant.

Chemo-GMT mice demonstrated similar levels of hepatic bile accumulation and LBP (Figure 6a,c) but elevated fibrosis (Figure 6b) compared to Vehicle-GMT counterparts. Hepatic transcription of *Cyp7a1*, *Cyp7b1*, and *Nr1h4* were lower in Chemo-GMT compared to Vehicle-GMT, while other genes altered in conventional Chemo were not differentially expressed (Figure 6d).

In the ileum, transcription of *Fgf15*, *Nr1h4*, *Vdr*, and *Cyp3a13* were lower in Chemo-GMT vs Vehicle-GMT, while *Scl10a2* and *Nr1i2* tended to be lower (Figure 6e). In the distal colon, only proliferation marker *Ki67* was higher in Chemo-GMT (Figure 6f).

## Discussion

Here-in, we provide evidence that paclitaxel chemotherapy-induced BAM is a multi-organ disorder involving the entire microbiota-enterohepatic axis, expanding the pathophysiology of BAM beyond enterotoxicity. We also highlight multiple compartments within the microbiota-hepatic axis as therapeutic targets to ameliorate chemotherapy-induced GI side effects, primarily gut barrier disruption and diminished gut microbial BA metabolism.

BAM induces malabsorption-associated weight loss in patients undergoing chemotherapy, attributed to BA-enhanced intestinal motility and reduced nutrient absorption.^36–38^ Similarly, we documented weight stagnation (during a linear phase of growth) in conjunction with increased fecal BA and water content, despite similar food consumption, decreased body temperature, and decreased locomotion. These data rule out metabolic inefficiency and activity-associated energy expenditure as sources of energy loss. While we were unable to quantify fecal energy loss, malabsorption is the most likely cause of the observed stunting and would mirror combined weight loss and BAM in patients undergoing chemotherapy.

Cholestasis is a reported side-effect in patients treated with chemotherapy including paclitaxel, although the underlying causes are not well-defined.^39^ As the liver is the primary site of xenobiotic (i.e. chemotherapeutic) detoxification and biliary elimination, hampering these processes would lead to bioaccumulation and hepatic toxicity. In this study, Chemo livers exhibited multiple signs of cholestasis and impaired xenobiotic metabolism as indicated by bile accumulation, fibrosis, diminished BA synthesis, and impaired expression of the primary enzyme in paclitaxel detoxification, *Cyp3a13*. Hepatic BA accumulation induces negative feedback on the classic (*Cyp7a1*) and alternative (*Cyp7b1*) BA synthesis pathways via Nr1h4 and Gpbar1 respectively.^40,41^ Notably, these feedback pathways do not seem to impair synthesis of TMCA, which is regulated by Cyp2c70.^41,42^ In line with this concept, hepatic expression of *Cyp2c70* was not different in Chemo mice (data not shown), and TMCA was elevated.

Our data also highlights the potential contribution of endotoxemia to chemotherapy-related cholestasis. The idea of LPS-induced cholestasis is not novel,^34,35^ but is underappreciated during cancer therapy where intestinal barrier function is routinely disrupted,^43^ promoting systemic LPS exposure that can induce cholestasis.^44^ Chemo mice exhibited indicators of intestinal barrier disruption and hepatic endotoxemia, including elevated hepatic LBP and reduced intestinal crypt depth and villus height. In particular, elevated hepatic LBP prompted investigation of hepatic LPS exposure *in vitro*. Indeed, LPS reduced expression of many genes involved in bile flow and BA, xenobiotic, and lipid metabolism as observed *in vivo*, demonstrating that hepatic endotoxemia may be clinically related to BAM in this population. Importantly, Chemo-GMT mice did not display most signs of LPS-induced cholestasis (increased bile stain, increased LBP, or reduced expression of genes reduced by LPS exposure). This suggests that changes in the gut microbiome alone are not sufficient to induce cholestasis, and that chemotherapy-induced systemic LPS exposure (most likely due to intestinal barrier disruption) is an important target for reducing BAM. Specifically, we propose that without chemotherapy-induced gut barrier disruption in Chemo-GMT mice there was reduced risk of translocation of gut microbial antigens such as LPS into circulation. Without higher hepatic LPS exposure, Chemo-GMT mice did not exhibit reduced expression of BA transporters nor increased bile staining.

BAM is primarily believed to occur in response to ileal enterotoxicity and mucositis.^45,46^ Enterotoxicity was evident in Chemo mice, which had diminished absorptive surface area in the small intestine. However, this was compounded by reduced expression of chief BA transporter *Slc10a2* (also known as *Asbt*) in the ileum. Chemo-induced cholestasis reduced luminal BA concentration in the proximal small intestine and reduced BA absorption in the distal ileum, presumably accounting for equivalent ileal luminal BA in Chemo and Vehicle. While we did not elucidate potential causes of *Slc10a2* downregulation, its expression adaptively decreases during cholestasis in both rodents and humans, and is related to circulating BA and bilirubin.^47,48^ Activation of BA receptors can also regulate expression of *Slc10a2*, and Chemo mice displayed reduced expression of multiple BA receptors throughout the small intestine.^49^ However, Chemo-GMT recapitulated nearly all of the same effects on ileal transcription witnessed in conventional Chemo mice, suggesting that changes in the small intestinal microbiota are also influential in chemotherapy-induced BAM. Similarly, other studies have noted that chemotherapy alters structure and function of the gut microbiome of mice and humans, and that germ-free rodents experience lower chemotherapy-induced enterotoxicity compared to their conventional counterparts.^50,51^ Lastly, expression of *Cyp3a13* was also diminished in the small intestine. Xenobiotic detoxification is believed to occur primarily in the liver, but this data indicates that intestinal detoxification of chemotherapeutics could be an interesting target to reduce enterotoxicity, although this requires further investigation.

BAM can lead to BA diarrhea (BAD) due to enhanced colonic BA exposure, exacerbating malabsorption and dehydration. We observed drastic changes in BA profiles and crypt morphology in the distal intestine of Chemo mice. Most prominently, the ratio of primary to secondary BA was elevated and marked by enhanced TMCA and diminished HDCA. Indeed, patients with BAD also have higher total fecal primary BA and higher primary BA to secondary BA ratio.^52^ Concurrently in our study, cecal crypt depth of Chemo mice shrank (as expected from enterotoxicity), but surprisingly distal colonic crypt depth grew concordantly with proliferation marker Ki-67. Crypt hyperplasia is problematic in that uncontrolled renewal of the intestinal epithelium leads to a higher proportion of poorly differentiated colonocytes, thus hampered secretory and absorptive functions. BA influence colonic motility and colonocyte proliferation conversely through activation of Nr1h4 or Gpbar1. Loss of Nr1h4 function promotes BAD, its activation suppresses colonocyte proliferation, and its expression was lower in the small intestine (but not the colon) of Chemo mice.^53,54^ Conversely, Gpbar1 activation stimulates colonic motility and colonocyte proliferation, and was more highly expressed in Chemo mice.^55^ *In-vitro*, TMCA did not alter expression of *Ki67* or *Gpbar1* in colonoids, while HDCA dampened *Ki-67* and *Gpbar1* expression. Chemo-GMT also diminished expression of *Nr1h4* in the ileum and enhanced distal colonic expression of *Ki-67*, further implicating microbial metabolites as key regulators of BAM pathophysiology. While HDCA is a dual Nr1h4 and Gpbar1 agonist,^56^ HDCA’s anti-proliferative effect in colonocytes was recently demonstrated to be Nr1h4-dependent in porcine colonoids, consistent with our study.^57^ Finally, although BA composition is somewhat different in rodents vs humans due to differences in primary bile acid synthesis (muricholic acids dominating in rodents vs cholic and chenodeoxycholic acids in humans) and gut microbiota composition, the overarching phenomena observed here in mice are translatable to humans receiving chemotherapy. As mentioned, BAD is associated with a higher primary to secondary bile acid ratio, and secondary BA common in humans like deoxycholic acid (which can be derived from cholic acid) also exert antiproliferative effects on the intestinal epithelium.^52,58^ Altogether, this suggests that therapies enhancing both Nr1h4 activation and microbial BA metabolism may be effective in ameliorating BAD.

BAM and reduced secondary BA concentrations in Chemo mice coincided with altered microbiota in every segment of the intestine. Taxonomic differences (primarily the phylum Firmicutes) increased from duodenum to ileum, then decreased from ileum to distal colon. Relative abundance of *Lactobacillus*, a genus recognized for its ability to tolerate and metabolize BA in the intestinal environment, was altered across most segments.^59^ *Lactobacillus* was inversely related to *Clostridium cluster IV* and *Lachnospiraceae NK4A136*. The ratio of these bacterial relative abundances was inversely related to TMCA:HDCA, implying that relative increases in *Lactobacillus* and decreases in *Clostridium cluster IV* and *L. NK4A136* occur in tandem with relative increases in TMCA and decreases in HDCA. Although potential HDCA metabolic pathways are understudied, one murine isolate (a Gram-positive rod) is documented to fully metabolize TMCA to HDCA.^60^ Notably, Bacteria in the genus *Clostridium* are well-recognized to metabolize primary BA to secondary BA and are mostly Gram positive rods.^61^ While considerably less is known about the family *Lachnospiraceae*, *L. NK4A136* is associated with altered lipid and BA metabolism in conjunction with elevated HDCA, consistent with our results.^62,63^ This places *Clostridium cluster IV* and/or *L. NK4A136* as prime candidates for this metabolic trait, but requires further investigation. *Bacteroides* (another BA-metabolizing genus) was depressed in the cecum and proximal colon where the ratio of primary to secondary BA was skewed by Chemo. Although it was not associated with TMCA:HDCA, *Bacteroides’* contribution to BA metabolism during chemotherapy should not be ruled out. A similar study providing moderate-dose melphalan chemotherapy to rats demonstrated reduced total circulating BA concentrations, reduced primary BA to secondary BA ratio, and reduced ileal absorptive surface area and *Slc10a2* expression in combination with reduced fecal microbial alpha diversity. This suggests than many of the underlying contributors to BAM could be similar between different chemotherapeutic classes.^64^

Several limitations to this study should be noted and addressed by future investigations. We investigated only female mice given that paclitaxel is primarily utilized in the treatment of female reproductive cancers (i.e. breast and ovarian). However, sexual dimorphism in BA-related phenotypes may exist between male and female animals treated with chemotherapy. Furthermore, these effects might not be easily extrapolated to other types of chemotherapy or multi-drug cocktails that have differing mechanisms of action and potential interactions. Fecal BA excretion was determined using a single sampling time point rather than an entire 24-hour period, which may provide a more-complete picture of BA excretion. Finally, since this study was not designed to mechanistically determine the causal series of events leading to these BA-related phenotypes, we cannot implicate hepatotoxicity of chemotherapy or LPS, enterotoxicity, or dysfunctional enteric microbial communities as the primary casual factor in the development of BAM.

In total, this work identifies several pathways by which paclitaxel chemotherapy influences BAM across the entire microbiota-enterohepatic axis in a segment-dependent manner. These data place host-microbe interactions as central targets for therapeutic interventions to ameliorate BAM, and prompt investigation of other cancer therapies for similar responses. We propose a working paradigm to be verified by future studies in which: 1) chemotherapy induces enterotoxicity – compromising intestinal barrier function, BA absorption, and enteric microbial populations, leading to 2) bacterial translocation, hepatic endotoxemia and cholestasis, 3) impaired BA absorption and microbial metabolism altering BA signaling in the colonic epithelium, which cumulatively 4) promote chemotherapy-induced GI symptoms. Therefore, development of therapies that leverage the microbiota to protect against enterotoxicity while promoting BA metabolism will likely enhance the quality of life and treatment outcomes for patients undergoing cancer therapy.

## Supplementary Material

Supplementary Tables.xlsx

## Abbreviations

BA: Bile Acids
BAM: Bile Acid Malabsorption
BAD: Bile Acid Diarrhea
CA: Cholic acid
CDCA: Chenodeoxycholic acid
Chemo: Chemotherapy
DCA: Deoxycholic acid
GI: Gastrointestinal
HCA: Hyocholic acid
HDCA: Hyodexoycholic acid
LBP: Lipopolysaccharide Binding Protein
LCA: Lithocholic acid
LPS: Lipopolysaccharide
TCA: Tauro-cholic acid
TCDCA: Tauro-chenodeoxycholic acid
TDCA: Tauro-deoxycholic acid
THDCA: Tauro-hyodeoxycholic acid
TLCA: Tauro-lithocholic acid
TMCA: Tauro-α-muricholic acid
TMCA: Tauro-β-muricholic acid
TUDCA: Tauro-ursodeoxycholic acid
UDCA: Ursodeoxycholic acid
αMCA: α-muricholic acid
βMCA: β-muricholic acid

## References

[1] Andreyev HJ, Davidson SE, Gillespie C, Allum WH, Swarbrick E, British Society of G. Practice guidance on the management of acute and chronic gastrointestinal problems arising as a result of treatment for cancer. Gut. 2012;61(2):179–20. doi:10.1136/gutjnl-2011-300563. 22057051 PMC3245898

[2] Oshima T, Miwa H. Gastrointestinal mucosal barrier function and diseases. J Gastroenterol. 2016;51(8):768–778. doi:10.1007/s00535-016-1207-z. 27048502

[3] Boussios S, Pentheroudakis G, Katsanos K, Pavlidis N. Systemic treatment-induced gastrointestinal toxicity: incidence, clinical presentation and management. Ann Gastroenterol. 2012;25(2):106–118. 24713845 PMC3959393

[4] Muls AC, Watson L, Shaw C, Andreyev HJN. Managing gastrointestinal symptoms after cancer treatment: a practical approach for gastroenterologists. Frontline Gastroenterol. 2013;4(1):57. doi:10.1136/flgastro-2012-100218. 28839701 PMC5369780

[5] Stein A, Voigt W, Jordan K. Review: chemotherapy-induced diarrhea: pathophysiology, frequency and guideline-based management. Ther Adv Med Oncol. 2010;2(1):51–63. doi:10.1177/1758834009355164. 21789126 PMC3126005

[6] Akbarali HI, Muchhala KH, Jessup DK, Cheatham S. Chemotherapy induced gastrointestinal toxicities. Adv Cancer Res. 2022;155:131–166. 35779873 10.1016/bs.acr.2022.02.007 PMC10033220

[7] Vijayvargiya P, Camilleri M. Current practice in the diagnosis of bile acid diarrhea. Gastroenterology. 2019;156(5):1233–1238. doi:10.1053/j.gastro.2018.11.069. 30844373

[8] Bowen JM, Gibson RJ, Coller JK, Blijlevens N, Bossi P, Al-Dasooqi N, Bateman EH, Chiang K, de Mooij C, Mayo B, et al. Systematic review of agents for the management of cancer treatment-related gastrointestinal mucositis and clinical practice guidelines. Supportive Care Cancer. 2019;27(10):4011–4022. doi:10.1007/s00520-019-04892-0. 31286233

[9] Hofmann AF. Bile acid malabsorption caused by Ileal Resection. Archiv Intern Med. 1972;130(4):597–605. doi:10.1001/archinte.1972.03650040121011. 4564853

[10] Dahlgren D, Sjöblom M, Hellström PM, Lennernäs H. Chemotherapeutics-induced intestinal mucositis: pathophysiology and potential treatment strategies. Front Pharmacol. 2021;12:12. doi:10.3389/fphar.2021.681417. PMC8129190 34017262

[11] Sun R, Xu C, Feng B, Gao X, Liu Z. Critical roles of bile acids in regulating intestinal mucosal immune responses. Therap Adv Gastroenterol. 2021;14:17562848211018098. doi:10.1177/17562848211018098. PMC8165529 34104213

[12] Mohanty I, Mannochio-Russo H, Schweer JV, El Abiead Y, Bittremieux W, Xing S, Schmid R, Zuffa S, Vasquez F, Muti VB, et al. The underappreciated diversity of bile acid modifications. Cell. 2024;187(7):1801–1818.e20. doi:10.1016/j.cell.2024.02.019. 38471500 PMC12248420

[13] Rowinsky EK, Donehower RC. Paclitaxel (Taxol). N Engl J Med. 1995;332(15):1004–1014. doi:10.1056/NEJM199504133321507. 7885406

[14] Loman BR, Jordan KR, Haynes B, Bailey MT, Pyter LM. Chemotherapy-induced neuroinflammation is associated with disrupted colonic and bacterial homeostasis in female mice. Sci Rep. 2019;9(1). doi:10.1038/s41598-019-52893-0. PMC6848141 31712703

[15] Gangloff A, Hsueh W-A, Kesner AL, Kiesewetter DO, Pio BS, Pegram MD, Beryt M, Townsend A, Czernin J, Phelps ME, et al. Estimation of paclitaxel biodistribution and uptake in human-derived xenografts in vivo with (^18^)F-fluoropaclitaxel. J Nucl Med. 2005;46(11):1866. 16269601

[16] Husain A, Aptaker L, Spriggs DR, Barakat RR. Gastrointestinal toxicity andClostridium DifficileDiarrhea in patients treated with paclitaxel-containing chemotherapy regimens. Gynecologic Oncol. 1998;71(1):104–107. doi:10.1006/gyno.1998.5158. 9784328

[17] Santos JC, Bever SR, Pereira-da-Silva G, Pyter LM. Tumor resection ameliorates tumor-induced suppression of neuroinflammatory and behavioral responses to an immune challenge in a cancer survivor model. Sci Rep. 2019;9(1):1–13. doi:10.1038/s41598-018-37186-2. 30679700 PMC6345941

[18] Si GLR, Yao P, Shi L. Rapid determination of bile acids in bile from various mammals by reversed-phase ultra-fast liquid chromatography. J Chromatographic Sci. 2015;53(7):1060–1065. doi:10.1093/chromsci/bmu167. 25520305

[19] Selwyn FP, Csanaky IL, Zhang Y, Klaassen CD. Importance of large intestine in regulating bile acids and glucagon-like peptide-1 in germ-free mice. Drug Metab Dispos. 2015;43(10):1544–1556. doi:10.1124/dmd.115.065276. 26199423 PMC4576674

[20] Bolyen E, Rideout JR, Dillon MR, Bokulich NA, Abnet CC, Al-Ghalith GA, Alexander H, Alm EJ, Arumugam M, Asnicar F, et al. Reproducible, interactive, scalable and extensible microbiome data science using QIIME 2. Nat Biotechnol. 2019;37(8):852–857. doi:10.1038/s41587-019-0209-9. 31341288 PMC7015180

[21] Quast C, Pruesse E, Yilmaz P, Gerken J, Schweer T, Yarza P, Peplies J, Glöckner FO. The SILVA ribosomal RNA gene database project: improved data processing and web-based tools. Nucleic Acids Res. 2013;41(D1):D590–D596. doi:10.1093/nar/gks1219. 23193283 PMC3531112

[22] Foster ZSL, Sharpton TJ, Grünwald NJ, Poisot TE, Poisot T. Metacoder: an R package for visualization and manipulation of community taxonomic diversity data. PLoS Comput Biol. 2017;13(2):e1005404. doi:10.1371/journal.pcbi.1005404. 28222096 PMC5340466

[23] Love MI, Huber W, Anders S. Moderated estimation of fold change and dispersion for rna-seq data with DESeq2. Genome Biol. 2014;15(12):550. doi:10.1186/s13059-014-0550-8. 25516281 PMC4302049

[24] Team RC. R: a language and environment for statistical computing. 2013.

[25] Fedarko MW, Martino C, Morton JT, González A, Rahman G, Marotz CA, Minich JJ, Allen EE, Knight R. Visualizing ’omic feature rankings and log-ratios using Qurro. NAR Genomics Bioinf. 2020;2(2):lqaa023. doi:10.1093/nargab/lqaa023. PMC7194218 32391521

[26] Morton JT, Marotz C, Washburne A, Silverman J, Zaramela LS, Edlund A, Zengler K, Knight R. Establishing microbial composition measurement standards with reference frames. Nat Commun. 2019;10(1):1–11. doi:10.1038/s41467-019-10656-5. 31222023 PMC6586903

[27] Kisielinski K, Willis S, Prescher A, Klosterhalfen B, Schumpelick V. A simple new method to calculate small intestine absorptive surface in the rat. Clin Exp Med. 2002;2(3):131–135. doi:10.1007/s102380200018. 12447610

[28] Berger CN, Crepin VF, Roumeliotis TI, Wright JC, Serafini N, Pevsner-Fischer M, Yu L, Elinav E, Di Santo JP, Choudhary JS, et al. The citrobacter rodentium type III secretion system effector EspO affects mucosal damage repair and antimicrobial responses. PloS Pathog. 2018;14(10):e1007406. doi:10.1371/journal.ppat.1007406. 30365535 PMC6221368

[29] Eissa N, Hussein H, Wang H, Rabbi MF, Bernstein CN, Ghia JE, Sly LM. Stability of reference genes for messenger RNA quantification by real-time PCR in mouse dextran sodium sulfate experimental colitis. PLoS One. 2016;11(5):e0156289. doi:10.1371/journal.pone.0156289. 27244258 PMC4886971

[30] Seglen PO. Chapter 4: Preparation of isolated rat liver cells. In: Prescott DM, editor. Methods in cell biology. Vol. XIII. New York, NY: Academic Press; 1976. p. 29–83. 10.1016/s0091-679x(08)61797-5 177845

[31] Innocenti F, Danesi R, Di Paolo A, Agen C, Nardini D, Bocci G, Del Tacca M. Plasma and tissue disposition of paclitaxel (taxol) after intraperitoneal administration in mice. Drug Metab Dispos: Biol Fate Chemicals. 1995;23(7):713–717. 7587959

[32] Grant CV, Loman BR, Bailey MT, Pyter LM. Manipulations of the gut microbiome alter chemotherapy-induced inflammation and behavioral side effects in female mice. Brain Behav Immun. 2021;95:401–412. doi:10.1016/j.bbi.2021.04.014. 33895287 PMC8461613

[33] Zhao L, Yang W, Chen Y, Huang F, Lu L, Lin C, Huang T, Ning Z, Zhai L, Zhong LLD, et al. A clostridia-rich microbiota enhances bile acid excretion in diarrhea-predominant irritable bowel syndrome. J Clin Investigation. 2020;130(1):438–450. doi:10.1172/JCI130976. PMC6934182 31815740

[34] Nishida M, Tamakuma S, Idei T, Mochizuki H. A research on the cholestasis caused by continuous endotoxemia. Nihon Geka Gakkai Zasshi. 1990;91(2):184–190. 2325602

[35] Razori MV, Maidagan PM, Ciriaci N, Andermatten RB, Barosso IR, Martín PL, Basiglio CL, Sánchez Pozzi EJ, Ruiz ML, Roma MG. Anticholestatic mechanisms of ursodeoxycholic acid in lipopolysaccharide-induced cholestasis. Biochemical Pharmacol. 2019;168:48–56. doi:10.1016/j.bcp.2019.06.009. 31202734

[36] Phillips SF. Absorption and secretion by the colon. Gastroenterology. 1969;56(5):966–971. doi:10.1016/S0016-5085(69)80101-0. 5782306

[37] Schmidt DR, Holmstrom SR, Fon Tacer K, Bookout AL, Kliewer SA, Mangelsdorf DJ. Regulation of bile acid synthesis by fat-soluble vitamins A and D. J Biol Chem. 2010;285(19):14486–14494. doi:10.1074/jbc.M110.116004. 20233723 PMC2863217

[38] Tough IR, Schwartz TW, Cox HM. Synthetic G protein-coupled bile acid receptor agonists and bile acids act via basolateral receptors in ileal and colonic mucosa. Neurogastroenterol Motil: Off J Eur Gastrointestinal Motil Soc. 2020;32(12):e13943. doi:10.1111/nmo.13943. 32656959

[39] Grigorian A, O’Brien CB. Hepatotoxicity secondary to chemotherapy. J Clin Transl Hepatol. 2014;2:95–102. 26357620 10.14218/JCTH.2014.00011 PMC4521265

[40] Chiang JY, Kimmel R, Weinberger C, Stroup D. Farnesoid X receptor responds to bile acids and represses cholesterol 7α-hydroxylase gene (CYP7A1) transcription. J Biol Chem. 2000;275(15):10918–10924. doi:10.1074/jbc.275.15.10918. 10753890

[41] Donepudi AC, Boehme S, Li F, Chiang JYL. G protein-coupled bile acid receptor plays a key role in bile acid metabolism and fasting-induced hepatic steatosis. Hepatology (Baltim, Md). 2017;65(3):813–827. doi:10.1002/hep.28707. PMC5195921 27351453

[42] Takahashi S, Fukami T, Masuo Y, Brocker CN, Xie C, Krausz KW, Wolf CR, Henderson CJ, Gonzalez FJ. Cyp2c70 is responsible for the species difference in bile acid metabolism between mice and humans. J Lipid Res. 2016;57(12):2130–2137. doi:10.1194/jlr.M071183. 27638959 PMC5321228

[43] Cinausero M, Aprile G, Ermacora P, Basile D, Vitale MG, Fanotto V, Parisi G, Calvetti L, Sonis ST. New frontiers in the pathobiology and treatment of cancer regimen-related mucosal injury. Front Pharmacol. 2017;8:354. doi:10.3389/fphar.2017.00354. 28642709 PMC5462992

[44] Assimakopoulos SF, Scopa CD, Vagianos CE. Pathophysiology of increased intestinal permeability in obstructive jaundice. World J Gastroenterol. 2007;13(48):6458. doi:10.3748/wjg.v13.i48.6458. 18161914 PMC4611283

[45] Camilleri M. Bile acid diarrhea: prevalence, pathogenesis, and therapy. Gut Liver. 2015;9(3):332–339. doi:10.5009/gnl14397. 25918262 PMC4413966

[46] Phillips F, Muls ACG, Lalji A, Andreyev HJN. Are bile acid malabsorption and bile acid diarrhoea important causes of loose stool complicating cancer therapy? Colorectal Dis. 2015;17(8):730–734. doi:10.1111/codi.12932. 25728737

[47] Hruz P, Zimmermann C, Gutmann H, Degen L, Beuers U, Terracciano L, Drewe J, Beglinger C. Adaptive regulation of the ileal apical sodium dependent bile acid transporter (ASBT) in patients with obstructive cholestasis. Gut. 2006;55(3):395–402. doi:10.1136/gut.2005.067389. 16150853 PMC1856080

[48] Sauer P, Stiehl A, Fitscher BA, Riedel HD, Benz C, Klöters-Plachky P, Stengelin S, Stremmel W, Kramer W. Downregulation of ileal bile acid absorption in bile-duct-ligated rats. J Hepatol. 2000;33(1):2–8. doi:10.1016/S0168-8278(00)80152-X. 10905579

[49] Yang N, Dong Y-Q, Jia G-X, Fan S-M, Li S-Z, Yang S-S, Li YB. ASBT(SLC10A2): a promising target for treatment of diseases and drug discovery. Biomed Pharmacother. 2020; 132. doi:10.1016/j.biopha.2020.110835. 33035828

[50] Chrysostomou D, Roberts LA, Marchesi JR, Kinross JM. Gut microbiota modulation of efficacy and toxicity of cancer chemotherapy and immunotherapy. Gastroenterology. 2023;164(2):198–213. doi:10.1053/j.gastro.2022.10.018. 36309208

[51] Rigby RJ, Carr J, Orgel K, King SL, Lund PK, Dekaney CM. Intestinal bacteria are necessary for doxorubicin-induced intestinal damage but not for doxorubicin-induced apoptosis. Gut Microbes. 2016;7(5):414–423. doi:10.1080/19490976.2016.1215806. 27459363 PMC5046166

[52] Min YW, Rezaie A, Pimentel M. Bile acid and gut microbiota in irritable bowel syndrome. J Neurogastroenterol Motil. 2022;28(4):549–561. doi:10.5056/jnm22129. 36250362 PMC9577585

[53] Dossa AY, Escobar O, Golden J, Frey MR, Ford HR, Gayer CP. Bile acids regulate intestinal cell proliferation by modulating EGFR and FXR signaling. Am J Physiol-Gastrointestinal Liver Physiol. 2016;310(2):G81–G. doi:10.1152/ajpgi.00065.2015. PMC4719061 26608185

[54] Ticho AL, Malhotra P, Dudeja PK, Gill RK, Alrefai WA. Bile acid receptors and gastrointestinal functions. Liver Res. 2019;3(1):31–39. doi:10.1016/j.livres.2019.01.001. 32368358 PMC7197881

[55] Ji C-G, Xie X-L, Yin J, Qi W, Chen L, Bai Y, Wang N, Zhao D-Q, Jiang X-Y, Jiang H-Q, et al. Bile acid receptor TGR5 overexpression is associated with decreased intestinal mucosal injury and epithelial cell proliferation in obstructive jaundice. Transl Res. 2017;182:88–102. doi:10.1016/j.trsl.2016.12.001. 28034761

[56] De Marino S, Carino A, Masullo D, Finamore C, Marchianò S, Cipriani S, Di Leva FS, Catalanotti B, Novellino E, Limongelli V, et al. Hyodeoxycholic acid derivatives as liver X receptor α and G-protein-coupled bile acid receptor agonists. Sci Rep. 2017;7(1):43290. doi:10.1038/srep43290. 28233865 PMC5324103

[57] Song M, Yang Q, Zhang F, Chen L, Su H, Yang X, He H, Liu F, Zheng J, Ling M, et al. Hyodeoxycholic acid (HDCA) suppresses intestinal epithelial cell proliferation through FXR-PI3K/AKT pathway, accompanied by alteration of bile acids metabolism profiles induced by gut bacteria. Faseb J. 2020;34(5):7103–7117. doi:10.1096/fj.201903244R. 32246800

[58] Dossa AY, Escobar O, Golden J, Frey MR, Ford HR, Gayer CP. Bile acids regulate intestinal cell proliferation by modulating EGFR and FXR signaling. Am J Physiol - Gastrointestinal Liver Physiol. 2016;310(2):G81–G92. doi:10.1152/ajpgi.00065.2015. PMC4719061 26608185

[59] O’Flaherty S, Briner Crawley A, Theriot CM, Barrangou R, Ellermeier CD. The lactobacillus bile salt hydrolase repertoire reveals niche-specific adaptation. mSphere. 2018;3(3):3. doi:10.1128/mSphere.00140-18. PMC5976879 29848760

[60] Eyssen HJ, De Pauw G, Van Eldere J. Formation of hyodeoxycholic acid from muricholic acid and hyocholic acid by an unidentified gram-positive rod termed HDCA-1 isolated from rat intestinal microflora. Appl Environ Microbiol. 1999;65(7):3158–3163. doi:10.1128/AEM.65.7.3158-3163.1999. 10388717 PMC91470

[61] Ridlon JM, Harris SC, Bhowmik S, Kang D-J, Hylemon PB. Consequences of bile salt biotransformations by intestinal bacteria. Gut Microbes. 2016;7(1):22–39. doi:10.1080/19490976.2015.1127483. 26939849 PMC4856454

[62] Caparrós-Martín JA, Lareu RR, Ramsay JP, Peplies J, Reen FJ, Headlam HA, Ward NC, Croft KD, Newsholme P, Hughes JD, et al. Statin therapy causes gut dysbiosis in mice through a pxr-dependent mechanism. Microbiome. 2017;5(1):1–15. doi:10.1186/s40168-017-0312-4. 28793934 PMC5550934

[63] Chen H, Zhou S, Li J, Huang X, Cheng J, Jiang X, Qin W, Liu Y, Liu A, Zhang Q, et al. Xyloglucan compounded inulin or arabinoxylan against glycometabolism disorder via different metabolic pathways: gut microbiota and bile acid receptor effects. J Funct Foods. 2020;74:104162. doi:10.1016/j.jff.2020.104162.

[64] Wardill HR, de Mooij CEM, da Silva Ferreira AR, van de Peppel IP, Havinga R, Harmsen HJM, Tissing WJE, Blijlevens NMA. Translational model of melphalan-induced gut toxicity reveals drug-host-microbe interactions that drive tissue injury and fever. Cancer Chemother Pharmacol. 2021;88(2):173–188. doi:10.1007/s00280-021-04273-7. 33877390 PMC8236460

